# Droopy Shoulder Syndrome: A Gateway to Thoracic Outlet Syndrome

**DOI:** 10.7759/cureus.62213

**Published:** 2024-06-12

**Authors:** Berrahal Lokman, Araj Aymane, Souhail Yachaoui, Ahmed Amine El Oumri

**Affiliations:** 1 Physical Medicine and Rehabilitation, Mohammed First University of Oujda, Oujda, MAR

**Keywords:** shoulder girdle strenghtening, brachial plexus, physical therapy, thoracic outlet syndrome, droopy shoulder syndrome

## Abstract

Droopy shoulder syndrome (DSS) manifests as discomfort or abnormal sensations in the neck, shoulder, chest, and upper limbs, resulting from tension on the brachial plexus caused by abnormally low shoulder positioning. This case report examines the presentation and management of a patient with DSS, a rare but crucial precursor to thoracic outlet syndrome (TOS). The patient, a 22-year-old male, presented with progressive pain and tingling in his left upper limb, shoulder, chest, and neck. Comprehensive examination and imaging studies led to a diagnosis of DSS. Physical therapy prevented progression to full-blown TOS, highlighting the importance of early recognition and intervention. This case underscores the diagnostic challenges and therapeutic strategies essential for managing this syndrome, preventing complications, and restoring patient function.

## Introduction

Droopy shoulder syndrome (DSS) and thoracic outlet syndrome (TOS) are intricate musculoskeletal disorders that are often interconnected [1,2]. DSS is characterized by progressive shoulder drooping accompanied by neurological symptoms and can serve as a precursor to TOS, which involves the compression of neurovascular structures within the thoracic outlet [1,2]. Recognizing the distinct features of each syndrome is essential for accurate diagnosis and effective intervention.

DSS manifests as a notable deviation in shoulder alignment and presents with symptoms such as numbness, tingling, and weakness in the upper extremity [1]. Early identification of DSS is crucial, as it can lead to TOS, a condition that can have debilitating consequences [2]. TOS includes a range of neurovascular complications due to compression within the thoracic outlet, affecting the brachial plexus, subclavian artery, or vein, and resulting in symptoms from pain and paresthesia to arterial insufficiency or venous thrombosis [2].

Given the overlapping symptoms and anatomical proximity of these conditions, the relationship between DSS and TOS warrants further exploration [1,2]. A thorough understanding of their definitions, clinical presentations, and underlying pathophysiology is necessary to navigate the diagnostic and therapeutic challenges they pose, ultimately optimizing patient outcomes.

This case presentation highlights the diagnostic process for TOS in a patient with a drooping shoulder, emphasizing the importance of early recognition and appropriate management [1,2].

## Case presentation

A 22-year-old male presented with a four-year history of persistent pain and tingling in his left upper arm, shoulder, neck, and chest. Symptoms were exacerbated by overhead arm elevation. Recently, he reported increasing numbness and tingling in his right arm and hand. His medical history was unremarkable, and he had no prior injuries, surgeries, or chronic conditions. The patient's body mass index (BMI) was 23.5, which is within the normal range.

The patient’s primary complaints included persistent pain and tingling in the left upper arm, shoulder, neck, and chest, worsening with overhead activities. He also reported increasing numbness and tingling in the right arm and hand, along with occasional weakness in the upper extremities, particularly during activities requiring arm elevation.

Physical examination revealed the absence of postural abnormality, bone deformity, and muscle imbalance. Palpation of the cervical spine, shoulder girdle, and supraclavicular area was normal, with no tenderness or reproduction of symptoms. The findings included a positive Ross sign on the left side, a positive Tinel sign in the left supraclavicular area, a positive Wright's test, and a positive Adson's test, all suggestive of neurological compression.

**Figure 1:**
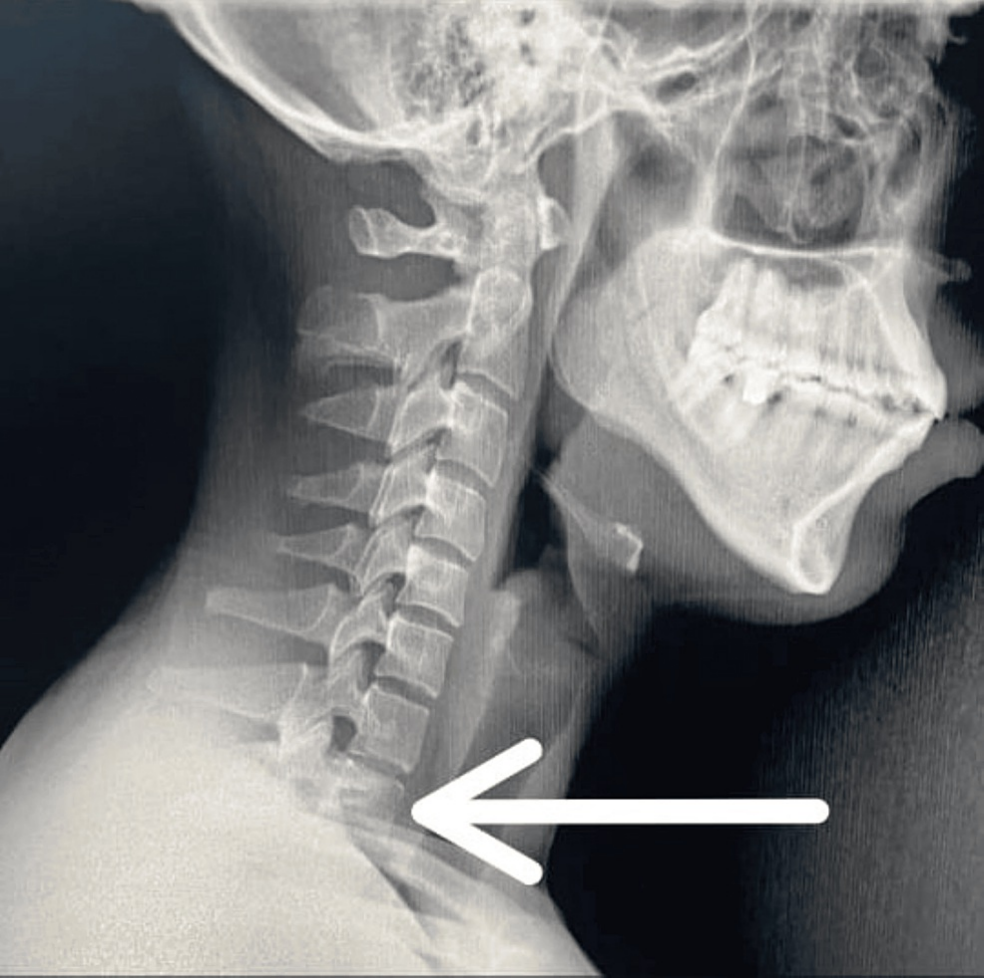
Visualization of the first thoracic vertebra (white arrow )

Negative clinical signs included negative Spurling's test, ruling out cervical radiculopathy; negative Lhermitte's sign, ruling out spinal cord involvement; and negative Phalen's test and Tinel's sign at the wrist, ruling out carpal tunnel syndrome. Cervical radiography was performed to rule out anatomical anomalies and visualized the first thoracic vertebra (Figure 1) (normally shoulders prevent visualization of the first thoracic vertebra) [3].

The patient was diagnosed with TOS secondary to DSS. A treatment plan was implemented with a frequency of two sessions per week, focusing on physical therapy. Strengthening exercises aimed to elevate and stabilize the shoulder girdle, focusing on the trapezius, levator scapulae, and rhomboid muscles. Exercises included shrugs and scapular retractions. Scapular stabilization exercises were designed to enhance the strength and coordination of the scapular muscles. These included wall push-ups, seated rows, and scapular push-ups. Stretching exercises aimed to alleviate tightness in the pectoral and scalene muscles. Pectoral stretches included the doorway stretch and the corner stretch. Scalene stretches included neck tilts and the scalene stretch. Postural correction involved education and exercises to promote correct posture, reducing strain on the neck and shoulders. Seated posture exercises and standing posture correction were emphasized. Manual therapy techniques addressed myofascial restrictions and improved mobility. Soft tissue mobilization included massage therapy and myofascial release. Joint mobilization focused on the thoracic spine and shoulder girdle. A structured home exercise program was provided to maintain and enhance the benefits achieved during therapy sessions. This included daily stretching and strengthening exercises, posture correction activities, and instructions on proper ergonomics to avoid exacerbating symptoms.

After 20 physical therapy sessions, the patient reported a significant reduction in pain and tingling, though some discomfort persisted during overhead activities. An additional 20 therapy sessions with a continued home exercise program were prescribed. Ten weeks later, the patient reported substantial improvement and symptom relief, underscoring the effectiveness of the extended physical therapy regimen and the importance of a multidisciplinary approach.

## Discussion

DSS is best diagnosed with a lateral cervical spine radiograph in suspected patients. On lateral radiographs of the cervical spine, it is unusual to visualize vertebral bodies inferior to the sixth or seventh cervical vertebrae. In the majority of patients, visualizing the seventh vertebra on a radiograph proves challenging, often requiring significant traction on both shoulders to achieve visibility. Contrastingly, individuals with DSS exhibit a distinct radiographic clarity, revealing not only the seventh cervical vertebra but also the first thoracic vertebra, with frequent visualization of a portion of the second thoracic vertebra [4].

DSS typically develops as follows: The patient, often a young woman, maybe mildly or moderately depressed. This leads to poor muscle tone, which is evident in a lack of facial expression and felt by the patient as painful shoulders. The drooping shoulders cause traction on the branches of the brachial plexus, resulting in pain [4].

Swift and Nichols outlined the following diagnostic criteria for identifying DSS, which include experiencing pain or paresthesia in the shoulder, neck, arm, forearm, or hand, physical characteristics such as long necks, low-set shoulders, and horizontally or downsloping clavicles, heightened symptoms upon palpation of the brachial plexus or passive downward traction of the arms, immediate alleviation of symptoms through passive shoulder elevation, absence of vascular symptoms, muscle atrophy, sensory deficits, or reflex alterations, normal results from nerve conduction studies, and visibility of the second thoracic vertebra or lower on radiographic imaging [3].

However, our patient diverged from the typical presentation described in the literature. Unlike previously published cases, our patient did not experience relief from symptoms with passive shoulder elevation. Furthermore, our patient did not undergo electromyography (EMG) to confirm nerve compression, given the evident symptom resolution achieved through physical therapy, rendering EMG unnecessary for immediate clinical management.

In our case, we diagnosed TOS based on the patient's clinical presentation and positive findings from maneuvers such as Ross, Tinel, Adson's, and Wright's tests. We subsequently ruled out congenital causes like cervical ribs, prolonged transverse processes, and exostosis of the first rib. Notably, the lateral radiograph displayed clear visibility of the first thoracic vertebra, prompting consideration of DSS as a potential underlying cause.

As the first step in our treatment approach, we prescribed a 20-session course of physical therapy. This regimen primarily emphasized strengthening the shoulder girdle, which consisted of several targeted exercises. Specifically, the exercises included scapular retraction and depression exercises such as seated rows and scapular squeezes. These exercises are supported by research demonstrating their effectiveness in improving scapular kinematics and shoulder stability [5]. Additionally, we focused on rotator cuff strengthening through internal and external rotation with resistance bands. Studies have shown these exercises to be essential for maintaining shoulder health and function [6]. To further enhance the shoulder girdle, we incorporated upper trapezius and serratus anterior activation exercises like shoulder shrugs and serratus punches. Activation of these muscles is critical for scapular stability and function [7].

Alongside the strengthening exercises, we integrated postural correction exercises into the regimen. These exercises aimed to improve the patient's overall posture, which is crucial for alleviating symptoms associated with DSS and TOS. The postural correction component included ergonomic education, such as proper desk setup and workplace modifications. Ergonomic guidelines from OSHA (Occupational Safety and Health Administration) support these interventions to prevent musculoskeletal disorders [8]. 

Extensive stretching routines were another critical aspect of the initial phase of therapy. These routines focused on key muscle groups that often contribute to the patient's symptoms. The stretching regimen included pectoral stretches like the corner stretch and doorway stretch. Stretching the pectoral muscles can alleviate tightness and improve posture [9]. Upper trapezius stretches involve gentle lateral neck flexion and levator scapulae stretches involve chin tucks and head tilts. These stretches are effective for relieving neck and shoulder tightness [10]. These exercises aimed to improve flexibility and reduce muscle tightness.

The significant improvement observed in the patient's symptoms during this initial phase affirmed the efficacy of our intervention. Encouraged by this progress, we decided to extend the therapy sessions. The extended sessions continued to yield consistently positive outcomes and introduced more advanced exercises to further enhance the patient's recovery.

In the advanced phase of physical therapy, we incorporated progressive resistance training to increase the intensity of strengthening exercises. The intensity of strengthening exercises was increased to build further strength and endurance [11]. Functional exercises were also introduced to mimic daily activities, ensuring the patient could translate their improvements into real-world scenarios. Functional training is critical for restoring daily life activities [12]. Dynamic postural training became a focus, with balance and proprioception exercises such as stability ball exercises and single-leg stands. These exercises improve coordination and stability [9]. We also included advanced postural drills, which involved dynamic postural corrections with added resistance to challenge and improve the patient's stability and coordination. Dynamic postural corrections with added resistance challenge and improve stability and coordination [6].

Enhanced stretching and mobility exercises were included in this phase to maintain and further improve flexibility. Dynamic stretching routines were implemented to incorporate movements that promote flexibility and functional range of motion. Dynamic stretching routines incorporate movements that promote flexibility and functional range of motion [11]. Thoracic spine mobility exercises, such as foam roller extensions and cat-cow stretches, were added to address any residual stiffness and enhance spinal mobility. These exercises address spinal stiffness and enhance mobility [7].

Neuromuscular re-education played a crucial role in the advanced phase. We introduced scapulothoracic rhythm training to improve coordination between the scapula and thorax, ensuring more efficient and effective movement patterns. Scapulothoracic rhythm training improves coordination between the scapula and thorax, ensuring efficient and effective movement patterns [5]. Additionally, biofeedback techniques were utilized to enhance muscle activation and postural awareness, further contributing to the patient's overall improvement. Biofeedback techniques enhance muscle activation and postural awareness, contributing to overall improvement [9].

The comprehensive approach and consistent positive outcomes highlight the importance of a structured rehabilitation protocol for managing DSS and TOS. The significant improvements in shoulder girdle strength, posture, and symptom reduction underscore the effectiveness of our treatment regimen. To maintain these gains, we emphasized the importance of ongoing strengthening and stretching exercises. The patient was provided with a maintenance program, including regularly scheduled exercises and periodic assessments, to adjust the exercise regimen as needed. Continued education on lifelong postural awareness and proper ergonomic practices was also reinforced to prevent recurrence.

In conclusion, the extended physical therapy sessions demonstrated substantial benefits, affirming the value of a comprehensive and patient-centered approach. Regular follow-ups and re-evaluations were scheduled to monitor progress and address any issues, ensuring long-term success and quality of life for the patient.

## Conclusions

This case highlights the intertwined nature of DSS and TOS, emphasizing early recognition and targeted intervention. Using diagnostic tools like lateral cervical spine radiographs and a multidisciplinary management approach, clinicians can effectively address structural anomalies and optimize patient outcomes. Continued research is essential to further understand these syndromes and improve diagnostic and therapeutic strategies.
